# Managing Dental Phobia in Children with the Use of Virtual Reality: A Systematic Review of the Current Literature

**DOI:** 10.3390/children10111763

**Published:** 2023-10-30

**Authors:** Alessio Rosa, Alberto Maria Pujia, Raffaella Docimo, Claudio Arcuri

**Affiliations:** 1 Department of Chemical Science and Technologies, Dentistry, University of Tor Vergata, 00133 Rome, Italy; 2 Department of Clinical Sciences and Translational Medicine, University of Rome “Tor Vergata”, 00133 Rome, Italy; 3 Department of Experimental Medicine and Surgery, University of Rome “Tor Vergata”, 00133 Rome, Italy

**Keywords:** dental phobia, pediatric dentistry, virtual reality, anxiety, behavior management, distraction systems, pain

## Abstract

Background: It is common today to encounter anxiety in patients facing dental treatment. Virtual reality (VR) is presented as a high-performing and innovative procedure because it can distract patients undergoing dental procedures or prepare them for such treatments. In addition, this meta-analysis has gathered evidence on VR and its ability to reduce dental anxiety in pediatric patients undergoing different treatments. Methods: The major Scopus, PubMed, EMBASE and Web of Science databases were searched for scientific articles published up to 2023. Studies in which VR was used for children and adults as a measure against anxiety during dental treatments were included. VR was defined as a three-dimensional place where patients experience a sense of immersion as they find themselves in attractive and interactive environments that detach them from reality. Anxiety and pain were examined and measured during dental treatments in which VR was used by comparing them with standard care situations. Results: Twenty-five studies were identified, of which eleven met the inclusion criteria. The effect of VR was studied mainly in the pediatric population as a distractive method. Only two studies (not significant) on an adult population were considered. Conclusions: this review shows that VR is an excellent distraction method that is effective in reducing anxiety before dental treatment; however, due to the few studies in this area, further research on VR as a tool to prepare patients for dental treatment is needed.

## 1. Introduction

Dental phobia is a prevalent issue among children that can significantly impact their oral health and overall well-being. It often leads to avoidance of dental visits, resulting in delayed or inadequate dental care, and the progression of oral health problems [1,2].

It is common practice today to witness anxiety in patients who have to undergo dental treatment. The literature offers a large number of studies on this phenomenon, which has been increasing disproportionately in recent years [3].

Several techniques have been proposed to better manage anxiety and pain in dental patients; pharmacologically, the use of anesthetics ensures that painless treatments can be performed, but anxiety and fear are often overlooked [4]. Virtual reality has been proposed as an innovative therapy because, although it is a difficult concept to define, it succeeds in detaching the patient from the thought of having to undergo dental treatment. The mechanism is a distractive type; the patient is immersed in a three-dimensional environment generated by computer technology that creates a sense of transportation and distraction [5,6].

The benefits of the therapeutic use of virtual reality for the management of dental anxiety and pain levels is described in multiple scientific studies and is becoming a commonly used palliative tool. Improved pain management and the reduced duration of perception of therapy has been found [7].

Conventional management techniques such as behavior guidance, distraction techniques, and pharmacological interventions have limitations in effectively addressing dental phobia in children [8,9]. However, recent advancements in technology have introduced virtual reality (VR) as a potential tool for managing dental phobia and improving the dental experience for children. This article aims to provide a comprehensive overview of the use of VR in managing dental phobia in children and its potential implications for pediatric dental practice [10].

VR refers to a computer-generated simulation that creates a multisensory, immersive environment, allowing users to interact with and experience a three-dimensional virtual world. It typically involves wearing a head-mounted display and may incorporate auditory and haptic feedback to enhance the sense of presence and immersion [11].

VR has been increasingly utilized in various healthcare fields, including pain management, mental health, rehabilitation, and medical education. Its immersive nature has shown promising results in reducing pain perception, anxiety, and distress, providing realistic simulations for training and therapy [12,13].

(1) VR provides an engaging and immersive experience that diverts attention away from the dental procedure, reducing anxiety and fear.
(2) Allows children to have a sense of control over their environment by offering interactive elements and customizable experiences, empowering them during dental visits.
(3) VR can create virtual scenarios that gradually expose children to dental procedures, helping them become more familiar and comfortable with dental settings, instruments, and sensations.
(4) VR can incorporate gamification elements and rewards, reinforcing positive behavior during dental procedures and promoting a positive dental experience.

The use of VR in managing dental phobia operates through several mechanisms.

The first is cognitive distraction. This mechanism diverts attention away from the dental procedure, VR reduces the focus on negative thoughts and fears, thereby alleviating anxiety [12,14].

The second is relaxation and a calming effect. The immersive nature of VR and the incorporation of calming environments can induce relaxation and create a sense of calmness during the dental visit.

Emotional regulation is another mechanism. VR can help children regulate their emotions by providing a safe space to experience and confront their fears in a controlled manner [7,15,16].

Multiple studies have demonstrated the effectiveness of VR in reducing dental anxiety in children. VR experiences have been shown to significantly decrease self-reported anxiety levels, the physiological markers of stress, and the behavioral indicators of anxiety during dental procedures [17,18,19].

VR holds significant promise as a therapeutic tool for managing dental phobia in children. By providing distraction, immersion, and desensitization, VR can effectively reduce dental anxiety, enhance positive dental experiences, improve cooperation, and facilitate behavior change. However, challenges such as ethical considerations, technical constraints, individual variations, and cost must be addressed to ensure the widespread implementation and accessibility of VR in pediatric dental practice [20,21]. Continued research, collaboration between dental professionals and VR developers, and the integration of VR using existing behavior management techniques can further optimize the use of VR and contribute to improved oral health outcomes for children with dental phobias.

This article aims to explore the use of VR as a therapeutic intervention in pediatric dentistry and its implications for managing dental phobia [22]. The meta-analysis, on the other hand, provides very useful information for understanding the real effect of a treatment or intervention in a specific group of patients and the accuracy of the estimation of the effect.

## 2. Materials and Methods

**Figure 1:**
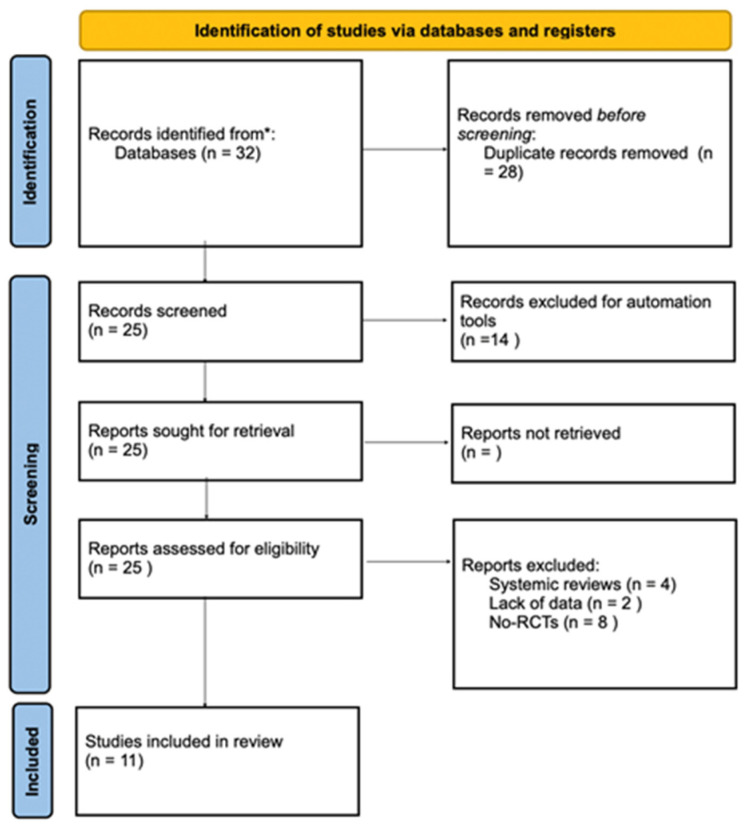
Search strategy flowchart of Database examined: * Scopus, Pubmed, Embase, Web of science.

This study followed the Preferred Reporting Items for Systematic Review and Meta-Analyses (PRISMA) statement (Figure 1). The primary research question was captured in the PICO (Population, Intervention, Comparison, Outcomes) format. Therefore, the aim of this systematic review was to answer the focused question: “Can the use of VR as a therapeutic treatment (I) reduce anxiety (O) in pediatric patients <18 years (P) undergoing dental procedures in comparison with adult patients (C)?”

Inclusion and exclusion criteria were defined by the authors before the study began. Studies measuring the effect of VR as a tool to reduce anxiety in pediatric patients aged ≤18 years undergoing dental procedures were included. VR was defined by the authors as a fully distractive three-dimensional computer-generated location displayed in stereoscopic vision on a display, often glasses, mounted on the head of the patient facing treatment (HMD). Studies that used 360-degree, non-computer-generated videos displayed on monitors were also considered eligible. Studies included in the meta-analysis had to have these requirements: a mean score for pain or anxiety during the procedure and a measure of distraction for both the intervention and control groups.

### 2.1. Exclusion Criteria

Reviews, meta-analyses, single-case studies, dissertations, conference papers, and abstracts were excluded.

### 2.2. Search Strategy

Two independent reviewers (A.R.) and (A.P.) performed a three-step screening procedure of all selected studies. First, the titles were screened to eliminate inappropriate studies. Then, all abstracts were reviewed, and only the full text of selected studies only was read. An exhaustive search of the following electronic databases was conducted on PubMed, Scopus, and Web of Science. Terms used for the research were virtual reality AND pediatric dentistry (“virtual reality” (MeSH terms) OR (“virtual” (all fields) AND “reality” (all fields)) OR “virtual reality” (all fields)) AND (“paediatric dentistry” (all fields) OR “pediatric dentistry” (MeSH terms)) OR (“pediatric” (all fields) AND “dentistry” (all fields)) OR “pediatric dentistry” (all fields)) (Table 1).

### 2.3. Selection Criteria and Data Extraction

Figure 1 shows a detailed overview of the study selection process. The search produced 25 articles. Two authors (A.R. and A.P.) independently evaluated the identified studies for compliance with inclusion and exclusion criteria. Discrepancies (2%) were discussed until an unambiguous consensus was reached. Based on title and abstract, 11 of the 25 studies were included. Most of these studies were excluded because they did not use VR or did not provide accurate and relevant data. Another reason was the lack of subjects aged <18 years. The final 11 studies were included. The MDAS and VAS questionnaire for assessing state anxiety were shown and explained to all children and their pre-treatment anxiety levels. The children were subsequently prepared for dental care. Each questionnaire was submitted to the patient to measure the degree of anxiety and pain during dental treatment, using conventional behavior management techniques (such as informal conversation, tell–show–do, mechanical distraction, game use, etc.).

### 2.4. Quality Assessment 

**Figure 2:**
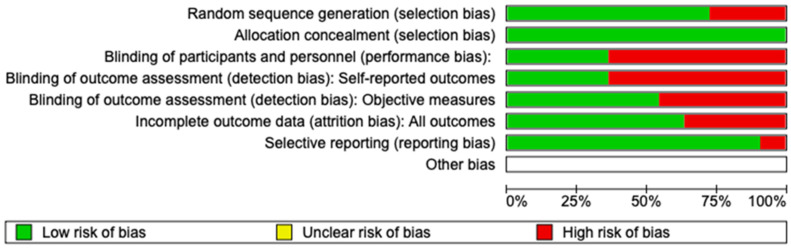
Risk of bias.

Two reviewers (A.P. and A.R.) assessed the risk of bias using version 2 of the Cochrane risk-of-bias tool for randomized trials (RoB 2). Any disagreement was discussed until a consensus was reached with the help of a third reviewer (R.D.) (Figure 2).

### 2.5. Statistical Analysis 

We performed the pooled analysis using Review Manager, version 5.2.8 (Cochrane Collaboration, Copenhagen, Denmark; 2014). We measured the risk ratio (RR) between the two groups (differences in pain when using or not using VR). Heterogeneity among studies was evaluated using the Higgins index (*I*^2^) and the chi-square test was classified as follows: low heterogeneity (<30%), medium heterogeneity (30–60%), and high heterogeneity (>60%). 

## 3. Results

### 3.1. Characteristics of the Studies

Pain levels in four studies were assessed, two on children [9,18,23] and two on adults [24,25]; anxiety levels were assessed in three studies, two on adults [15,21] and one on children [26]; and anxiety and pain together were assessed in seven studies, five on children [26,27,28,29,30] and two on adults [11,12] (Table 2).

### 3.2. VR and Anxiety Management

**Figure 3:**
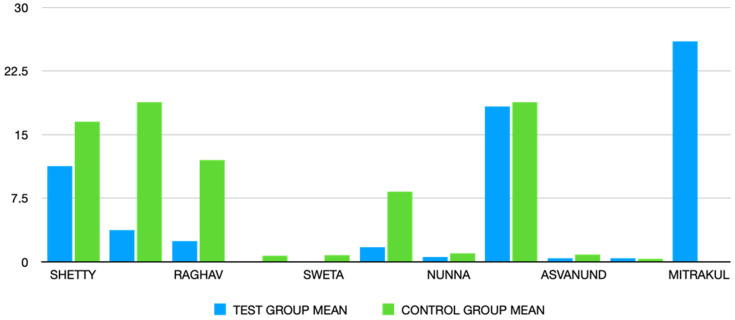
Statistical graph of mean anxiety measurements in all studies included.

Regarding anxiety, the composite measure was not significant, either for the whole (children + adults) (*p* = 0.302) or children (*p* = 0.243) and adults (*p* = 0.567) separately (Figure 3). Heterogeneity appears to be moderate. Anxiety was measured in two studies featuring virtual reality via visual analogue scale or the modified child dental anxiety scale (VAS and MDAS) [31].

### 3.3. VR and Pain Management

Regarding pain, VR provided significant protection for children (SMD = −0.82, i.e., a substantial effect according to Cohen’s scale [27]) but not for adults, as the 95% confidence interval (CI) includes the null value 0. However, this is based on only two studies.

Aminabadi et al. used the MDAS and consolability scale (FLACC), with these authors finding a statistically significant (*p* < 0.001) decrease in anxiety scores with the use of VR distraction [26].

### 3.4. Meta-Analysis

**Figure 4:**
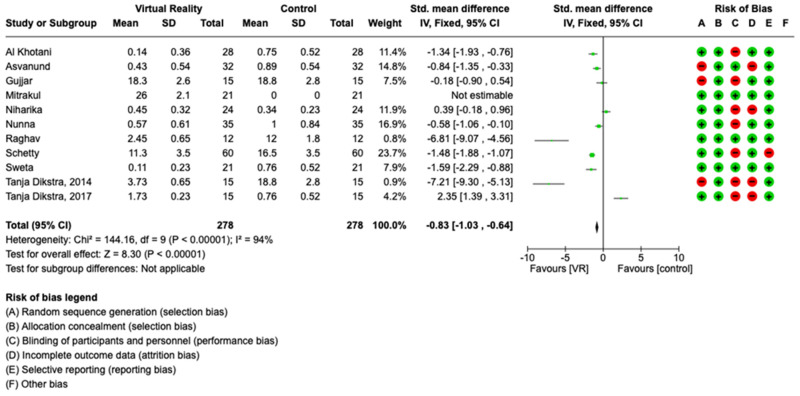
Meta-analysis of studies included.

**Figure 5:**
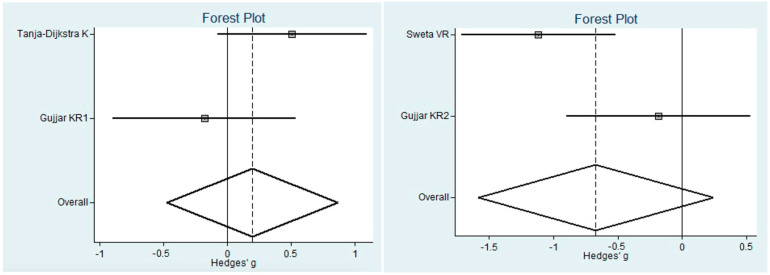
Forest plot for pain in children and pain in adults.

The meta-analysis was conducted through a random model effect because of the high heterogeneity (*I*^2^ = 94%) among the eleven included studies that compared the prevalent differences in pain as measured using the VAS scale and the MDAS scale. The chosen outcome of comparison in the eleven studies was the differences in pain between patients that used VR or not. The overall effect, reported in the forest plot (Figure 4), revealed that subjects exposed to VR had a lower sense of pain during the dental procedure than controls not exposed to VR (std mean −0.83; 95% CI: −1.03; −0.64; Z = 8.30; *p* = 0.00001) (Figure 5).

### 3.5. Quality Assessment and Risk of Bias

**Figure 6:**
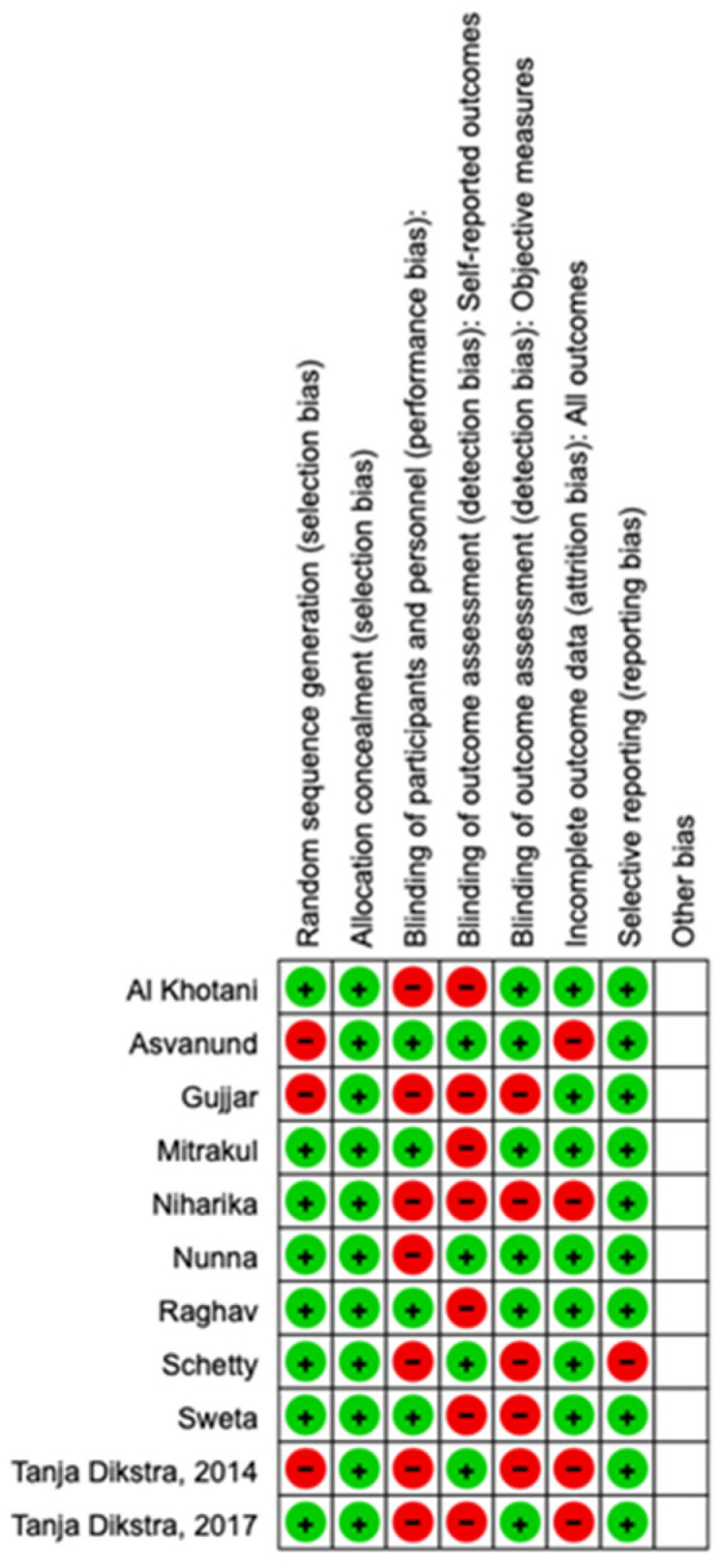
Quality assessment.

Using RoB 2, the risk of bias among the studies analyzed was estimated and is reported in Figure 2 and Figure 6. Regarding the randomization process, 75% of studies had a high risk of bias. Regarding allocation concealment, 100% of studies had a low risk of bias. Only 25% of studies excluded performance bias, and 25% reported all outcome data; however, 85% of the included studies presented a low risk of reporting bias.

## 4. Discussion

Dental procedures can often cause pain and anxiety, and children are especially sensitive to these two phenomena [2].

In the past, the main focus of managing patient pain and anxiety was centered on pharmacological treatments, while the literature published in the last decade has increasingly focused on non-pharmacological techniques. One cognitive behavioral strategy is called distraction, a technique based on the notion of a human being’s limited attention span. Distraction techniques range from passive to active interventions, with the belief that the more interactive the distraction technique, involving visual, auditory, and tactile stimuli, the greater the potential for distraction from pain. The management of dental phobia in children is a complex challenge that requires innovative approaches to alleviate the fear and anxiety associated with dental procedures [9,19].

Although most of the studies included in this meta-analysis are focused on pediatric dental patients, it should be noted that dental fear and anxiety affects 15–20% of the world’s population. It is precisely from this pathology that a refusal is generated towards dental care that pushes patients to avoid even the most basic treatments such as simple professional hygiene [32].

There are many works in the literature that describe the use of VR; however, this systematic review with meta-analysis is focused specifically on measuring the effectiveness of VR in patients undergoing dental care, especially if they are afraid of dentistry. This meta-analysis is based on a sample of eleven studies included after careful research in the literature: five studies measured anxiety, three studies examined the pain experienced during dental care, and three studies covered both experiences [30].

VR has emerged as a promising therapeutic tool in pediatric dentistry, offering a unique and immersive experience that can effectively address dental phobia in children. The use of VR provides distraction, immersion, and desensitization, which helps reduce dental anxiety, enhance positive dental experiences, improve cooperation, and facilitate behavior change [33,34].

VR has proven effective in reducing dental anxiety in children, as highlighted by numerous studies. By taking the focus away from the dental procedure, VR helps alleviate the fear and negative thoughts associated with dental visits [22,23,24]. The interactive and immersive nature of VR also promotes a sense of control, allowing children to actively participate and feel empowered during the dental experience. Furthermore, VR facilitates desensitization by gradually exposing children to dental stimuli, helping them to become familiar and comfortable with the dental environment, tools, and sensations [35,36,37,38,39].

In addition to reducing anxiety, VR enhances positive dental experiences for children. By creating an engaging and pleasant environment, VR can transform a dental visit into a positive and engaging activity [28,29].

Incorporating elements of gamification and rewards within the VR experience reinforces positive behavior, promotes cooperation, and fosters a favorable attitude towards dental care. These positive experiences have the potential to reshape children’s perceptions of dental visits and contribute to long-term oral health habits.

Despite the significant advantages of VR in the management of dental phobia, several challenges and limitations need to be addressed. Ethical considerations, such as informed consent and age appropriateness, are critical to ensuring the responsible use of VR technology [36,40].

The results of the present review showed that VR interventions for pain and anxiety are more effective when applied to pediatric subjects rather than adults. One possible explanation can be found in the fact that children experience higher levels of anxiety during treatment. Another possible explanation, however, is that they are more attracted to and involved in VR than adults, as they become trapped and fascinated by magic thinking [41].

Because the relationship between age and the effectiveness of VR on pain or anxiety might be different within each study, the relationship shown between age and the effectiveness of VR in the meta-regression might not represent the true scientific relationship for the fallacy phenomenon [42].

Another interesting study showed how the application of VR during the primary teeth extraction procedures of children, always under local anesthesia, can reduce anxiety and the feeling of pain. But the use of VR is not sufficiently adequate for children with severe anxiety [39]. The use of VR helmets in local anesthesia and primary teeth extraction can significantly reduce dental anxiety and pain perception in children without the occurrence of simulator sickness. But it is not adequate to help children with severe anxiety complete their treatment [43,44].

Many studies show that virtual reality is more effective in reducing pain and anxiety than usual (CAU) treatment. It remains difficult to separate the added value of virtual reality from other forms of distraction, such as watching a television cartoon, because CAU is often not well defined in its distractive form. The quality of the effects studied suggested that VR distraction is perhaps more effective than CAU distraction during medical procedures. For example, a Cochrane review found an effect size of 0.21 for the impact of mechanical distraction (toys, colors) on self-reported pain during dental procedures. Similarly, a meta-analysis of studies on music therapy as a distraction during different types of medical procedures found that it significantly reduced pain and anxiety with an effect size of 0.22 of VR compared to other distractive techniques [45]. However, other effect dimensions are needed to advance VR as a preparation tool for medical procedures compared to other forms of preparatory interventions to reduce pain and anxiety [46].

Furthermore, the quality scores of studies that were published after the review by Eijlers [2] are higher, suggesting that the quality of studies has greatly improved in recent years, thanks to applications aimed at the use of VR in private dental practices.

The studies of this systematic review and meta-analysis are different in statistical quality. Most used randomization by clearly establishing inclusion and exclusion criteria. However, the type of distraction technique used was not always provided [47]. In addition, few studies have focused on the patient’s sensitivity to VR; an important element to be analyzed is the immersion of the patient in VR, which is influenced by the interaction of the subject with the virtual environment through virtual physical movements such as position change, orientation change, and point of view [43]. Non-VR content creates less immersion because the user is limited in all these virtual movements. Regarding this difference, it has been hypothesized that the greater the immersion of the patient, the greater the reduction in the pain because the patient is more distracted and therefore paying little attention to the perception of pain [48,49]. 

Although some studies included questions about the state of mind of patients during the immersive stage, it was difficult to analyze this phenomenon. Another limit is the immobility of the head that the patients must often maintain during dental treatments. However, the alleged superiority of VR over audiovisual spectacles and non-VR content with regard to effectiveness in medical care has yet to be demonstrated [50]. Therefore, the role of immersion should be at the heart of future research.

Technical constraints, including image resolution, motion sickness and latency, should be continuously improved to optimize the immersive experience. Individual variations in response to VR require personalized approaches to meet different preferences and needs. Additionally, the cost and accessibility of VR technology should be considered to ensure its widespread adoption in pediatric dental practices [1].

Looking to the future, standardization and guidelines for the use of VR in the management of dental phobia in children are essential to establish best practices and ensure consistency across dental environments. Integrating VR with existing behavior management techniques can improve their effectiveness and provide comprehensive solutions for managing dental phobia [42].

Long-term efficacy studies are needed to evaluate the persistence of anxiety reduction and the impact on oral health outcomes. Furthermore, tailoring VR experiences to individual preferences and needs can optimize the effectiveness of the intervention [50,51].

In conclusion, VR holds great promise as a valuable tool in the management of dental phobia in children. By addressing anxiety, enhancing positive experiences, and promoting behavior change, VR has the potential to transform the pediatric dental practice [36].

With continued research, technological advances, and collaborative efforts, VR can help improve oral health outcomes, promote lifelong dental care, and ensure positive dental experiences for children with dental phobia. The use of VR has the potential to transform a dental visit into a positive and fun experience for children.

By creating an engaging and interactive environment, VR can promote positive emotions, increase satisfaction, and foster a more favorable attitude towards dental care [36].

Children with dental phobia often exhibit non-cooperative behavior during dental procedures, making treatment challenging. VR has shown to improve cooperation and compliance by increasing engagement, reducing anxiety-related resistance, and facilitating a more relaxed and cooperative attitude towards treatment [42].

VR can serve as a valuable tool for desensitization, gradually exposing children to dental stimuli and procedures in a controlled manner. Through repeated exposure in the virtual environment, children can build resilience, reduce fear responses, and develop coping mechanisms, preparing them for real-life dental encounters [52,53].

There are some limitations on the use of VR. Ethical considerations must be addressed, particularly regarding informed consent, age appropriateness, and potential psychological impact [48,49].

The effectiveness of VR may be influenced by technical limitations, such as image resolution, motion sickness, latency, and the availability of suitable content. Ongoing advancements in VR technology are necessary to enhance the immersive experience and address these technical challenges [50,51].

These findings are paralleled by the work of Aitken and Sullivan, who showed that the use of VR distraction was effective in reducing state anxiety during dental treatment in children even without dental phobia. In Sullivan’s study, only two children were not totally distracted by VR, and these children were later excluded from the study [26,47]. Children may exhibit variations in their response to VR, with some requiring additional support or alternative strategies. Individualized approaches should be considered to accommodate different preferences, needs, and levels of dental phobia [6].

The implementation of VR in dental practices may involve initial costs, including hardware, software, and content development [54,55].

Ensuring the accessibility and affordability of VR technology is essential for widespread adoption and utilization in pediatric dental settings. Establishing standardized protocols and guidelines for the use of VR in managing dental phobia in children can ensure consistency, safety, and best practices across different dental settings [2].

Combining VR with existing behavior management techniques, such as tell–show–do, positive reinforcement, and non-pharmacological approaches, can enhance their efficacy and provide comprehensive solutions for dental phobia management [56].

Future studies, for example, could consider the use of eye-tracking systems and compare their effectiveness in reducing pain and discomfort compared to traditional VR during dental procedures [40,57]. Virtual reality gadgets with higher resolutions and wider field of views were found to be effective in significantly reducing thermal pain on the skin and the time spent thinking about the pain compared to low resolution and narrower field VR systems [15]. Additional potential areas of study in this field include the patient satisfaction and enjoyment with the virtual reality experience during dental visits [44].

Further research is needed to evaluate the long-term efficacy of VR interventions, including the persistence of anxiety reduction, the impact on oral health outcomes, and the potential for sustained behavior change in children with dental phobia [45,58].

Finally, VR presents itself as a promising tool that has until now been underestimated in dentistry. Virtual reality in dentistry has proven to be very effective but still has major limitations because it is necessary for the head to remain still during dental therapy. For this reason, high-quality studies are recommended to evaluate pre-operative VR or applications on new smartphones to prepare patients for dental care and local anesthesia procedures [31,41]. The current work found only a small number of articles in the databases included so the sensitivity of meta-analysis could be affected by various parameters including sample size, study design, and reporting standard. A systematic review should therefore be carried out with a targeted scope towards the use of VR in the dental field only; however, achieving this will require randomized controlled trials that involve the use of a virtual world for each individual dental treatment without differences in the ages of the population analyzed. This would improve statistical effectiveness and reliability. In order to obtain a concrete measurement of when VR affects anxiety and pain in dental treatments, it would be necessary to reduce the risk of uncertainty; eliminate confusing factors, such as the presence of parents during therapy; and establish a clear definition of the appropriate parameters to be measured [44]. Only in this way can the results be scientifically relevant and clinically applicable in support of daily dental practices.

Adapting VR experiences to individual preferences and needs can optimize the effectiveness of the intervention. Customizable content and interactive elements can foster interaction and promote a sense of ownership over the dental experience. In the world today, which is increasingly moving towards the use of artificial intelligence in all sectors, it would be beneficial to strengthen the current techniques of VR and, perhaps, to create a pre-established protocol of use of virtual immersion in patients who on their first visit already demonstrate limits during treatment [59,60].

## Figures and Tables

**Table 1:** Search strategy using MeSH terms.

*PubMed* virtual reality AND pediatric dentistry (“virtual reality” (MeSH terms) OR (“virtual” (all fields) AND “reality” (all fields)) OR “virtual reality” (all fields)) AND (“paediatric dentistry” (all fields) OR “pediatric dentistry” (MeSH terms) OR (“pediatric” (all fields) AND “dentistry” (all fields)) OR “pediatric dentistry” (all fields))
*Web of Science* (virtual reality) AND (pediatric dentistry) (all fields)
*Scopus* (virtual reality) AND (pediatric dentistry) TITLE ABS KEY

**Table 2:** Main characteristics of studies included (CHI: children; ADUL: adult; MDAS: modified dental anxiety scale; VAS: visual analog scale; FLACC (consolability scale); P = pain; DA = anxiety).

Age Group/Study	Scale for Pain and Dental Anxiety	Children Values	Mean ± SD	Adult Values	Mean ± SD
**7 Children + 2 Adults = 9 Total**		282 Total		284 Total	
Shetty—2CHI	MDAS = DA	*N* = 60	11.3 ± 3.5	*N* = 60	16.5 ± 3.5
Tanja Dikstra 2014—1 ADUL	MDAS = DA FLACC = P	*N* = 15	3.73 ± 0.65	*N* = 15	18.8 ± 2.8
Raghav 2016—1 CHI	VAS = P	*N* = 12	2.45 ± 0.65	*N* = 12	12 ± 1.8
Al Khotani—1 CHI	MDAS = DA VAS = P	*N* = 28	0.14 ± 0.36	*N* = 28	0.75 ± 0.52
Sweta—1 CHI	VAS = P	*N* = 21	0.11 ± 0.23	*N* = 21	0.76 ± 0.52
Tanja Dikstra 2017—1 ADU	MDAS = DA FLACC = P	*N* = 15	1.73 ± 0.23	*N* = 15	8.23 ± 2.34
Nunna—1 CHI	VAS = DA	*N* = 35	0.57 ± 0.61	*N* = 35	1 ± 0.84
Gujjar—1 CHI	MDAS = DA	*N* = 15	18.3 ± 2.6	*N* = 15	18.8 ± 2.8
Asvanund	VAS = P/DA	*N* = 32	0.43 ± 0.54	*N* = 32	0.89 ± 0.54
Niharika	VAS = P	*N* = 24	0.45 ± 0.32	*N* = 24	0.34 ± 0.23
Mitrakul	MDAS = P/DA	*N* = 21	26 ± 9.1	*N* = 21	0.1 ± 0.2

## Data Availability

The data from the present study can be obtained upon reasonable request from the corresponding author.
